# Supplementary figures and images for: CYP2B6*6 single nucleotide polymorphism among patients with uncomplicated malaria in Adjumani district, Uganda: Implications on efficacy of artemether-lumefantrine
Source: PLoS One. 2025 Aug 1;20(8):e0322918. doi: 10.1371/journal.pone.0322918 (PMC12316228; doi:10.1371/journal.pone.0322918)

Image 1

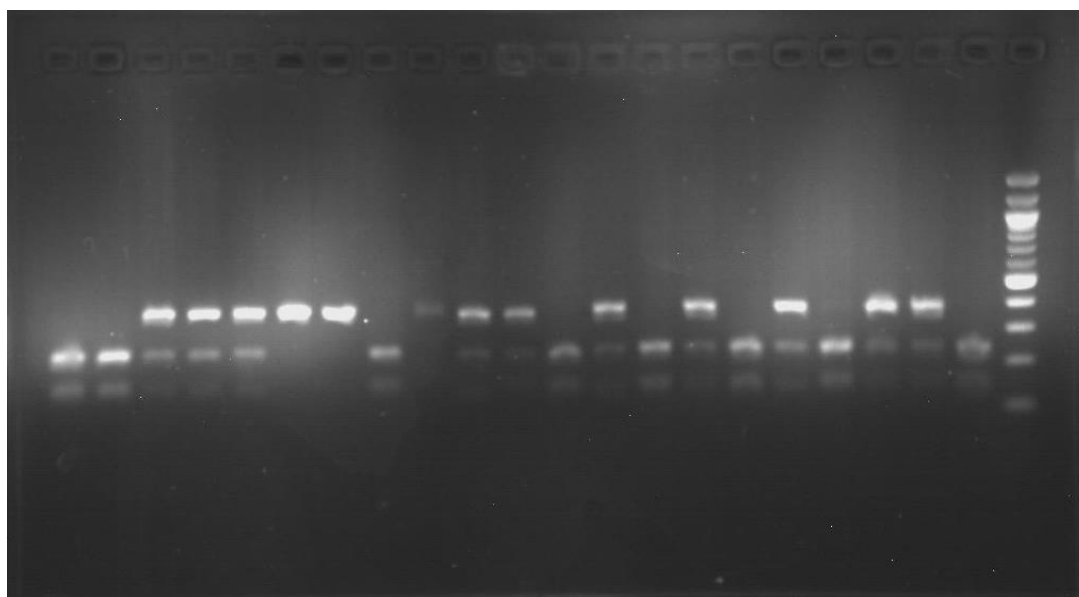

Image 2

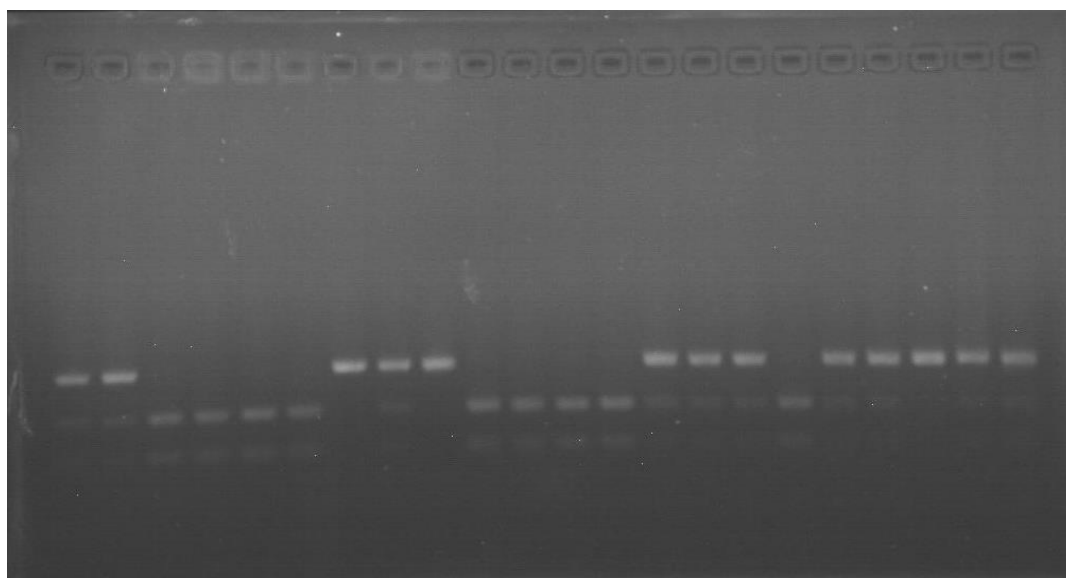

Image 3

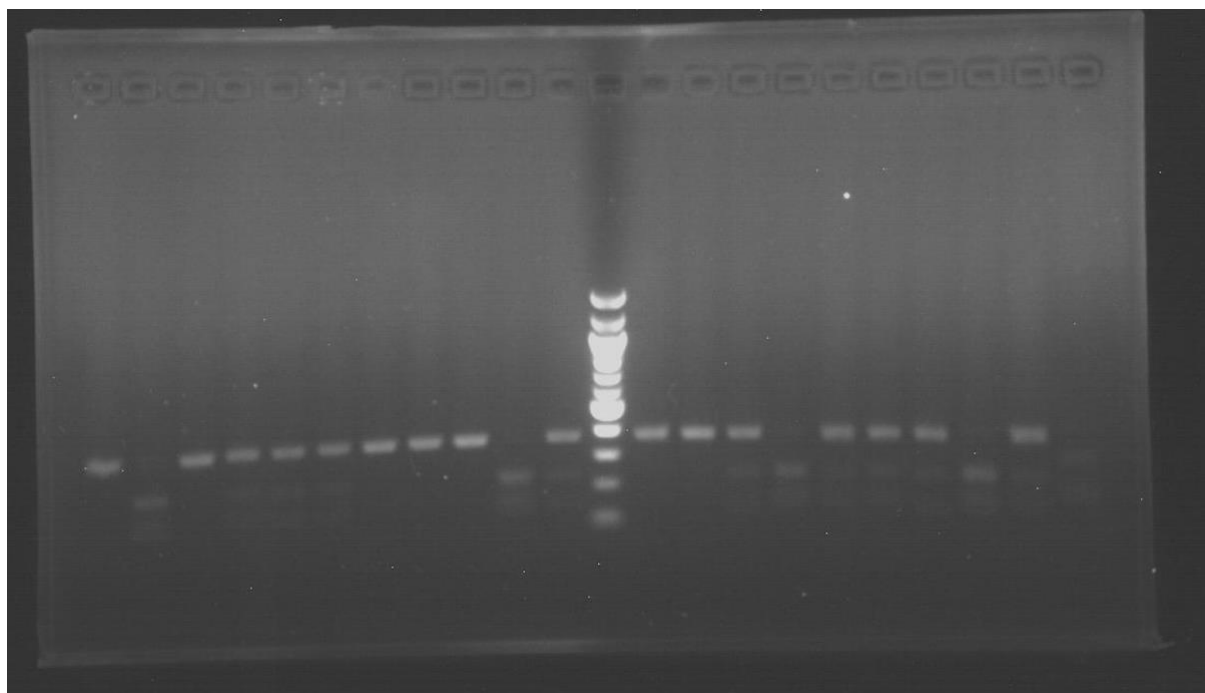

Image 4

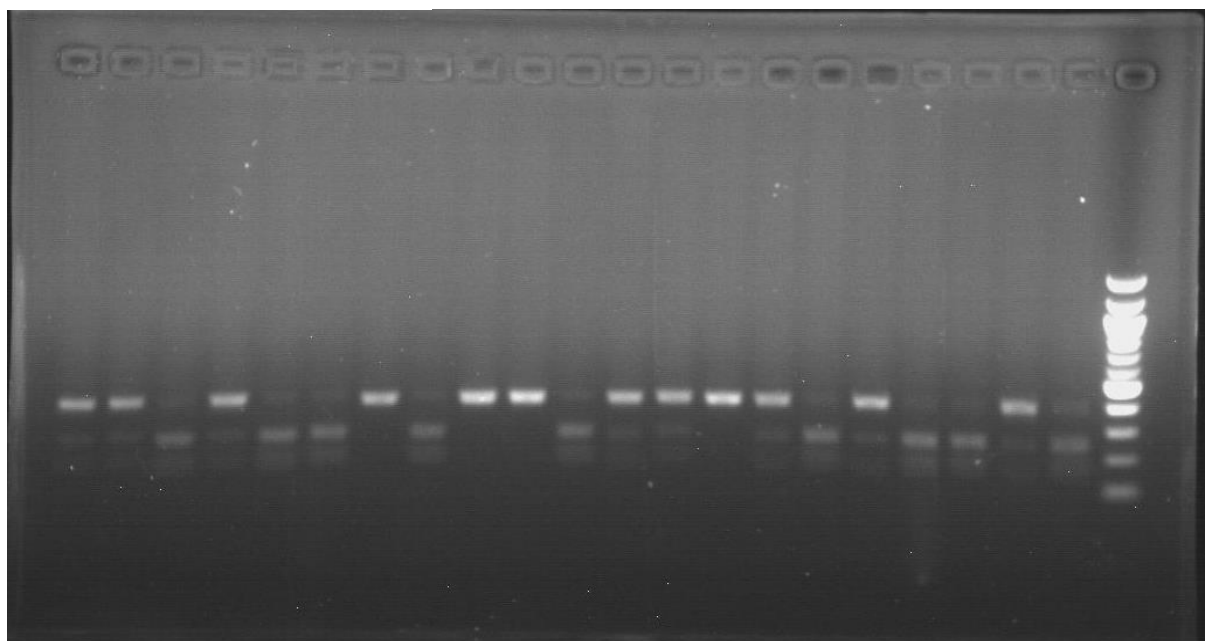

Image 5

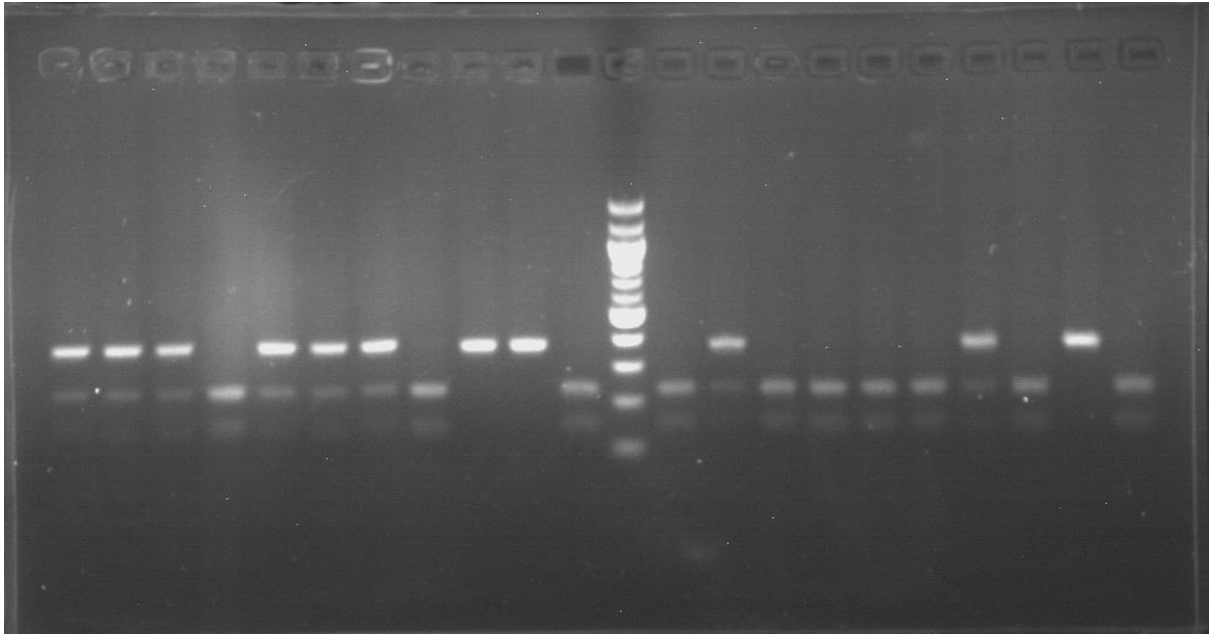

Image 6

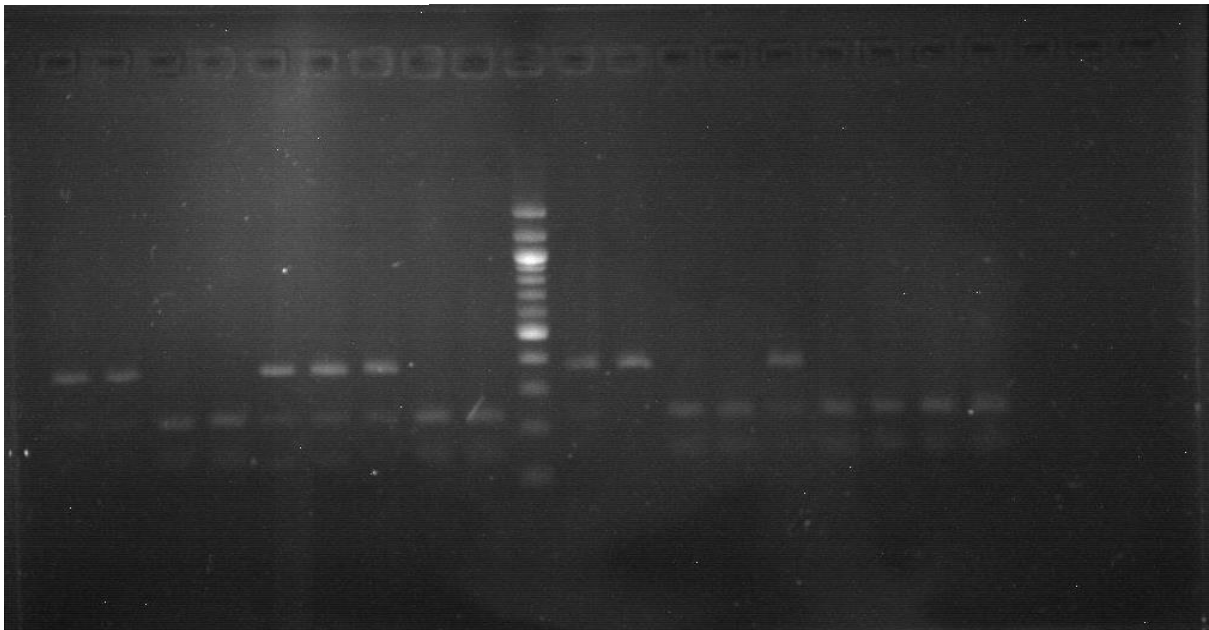

Supplement: S1 Fig — (PDF) [file pone.0322918.s001.pdf]
